# Growth pattern of uterine leiomyoma along pregnancy

**DOI:** 10.1186/s12905-019-0803-5

**Published:** 2019-07-22

**Authors:** Henry Hillel Chill, Gilad Karavani, Talya Rachmani, Uri Dior, Ofer Tadmor, Asher Shushan

**Affiliations:** 1 Department of Obstetrics and Gynecology, Hadassah Hebrew University Medical Center, Ein-Kerem, PO Box 12000, 91120 Jerusalem, Israel; 2 Maccabi Health Services, Jerusalem, Israel

**Keywords:** Uterine leiomyoma, Uterine fibroid, Pregnancy, Ultrasound, Complicated pregnancy

## Abstract

**Background:**

Uterine leiomyomas are often discovered during early pregnancy and in most cases will have no effect on pregnancy outcomes. However, in rare cases uterine leiomyomas may lead to obstetric complications. The aim of the study was to evaluate rate of uterine leiomyoma growth in the 3 trimesters of pregnancy.

**Methods:**

We conducted a retrospective cohort study. Included were women who were diagnosed with uterine leiomyoma during pregnancy and had at least two sonographic measurements in different trimesters. Data regarding leiomyoma growth, recorded by ultrasound examination, during 1st 2nd and 3rd trimesters were collected from electronic patient records.

**Results:**

Two-hundred forty-eight uterine leiomyomas were included in the study. Leiomyoma area increased substantially in size between the 1st and 2nd trimesters (54.5% ± 75.9%, *p* = .007) and to a lesser degree between the 2nd and 3rd trimesters (17.9% ± 59.7%, NS). Evaluation of the change in size throughout the pregnancy – between 1st and 3rd trimesters revealed a significant increase of 95.9% ± 191.3% (*p* < .001). There was no significant growth of the leiomyomas between the 2nd and 3rd trimesters.

**Conclusions:**

Uterine leiomyomas tend to grow substantially during the 1st trimester of pregnancy. This trend is attenuated later with minimal growth towards the end of gestation.

## Background

Uterine leiomyomas have challenged the scientific community for the past two centuries and yet the pathogenesis of uterine leiomyoma growth is still unclear. Estrogen and progesterone have been implicated as important instigators of leiomyoma growth together with cytokines, growth factors, chemokines and other regulatory proteins [1–4]. Recent studies have pointed towards Human Chorionic Gonadotropin (HCG) as a major contributor towards Leiomyoma growth [5, 6].

Uterine leiomyomas are often an incidental finding discovered during an ultrasound (US) examination performed in early pregnancy [7]. In pregnancy, their prevalence is estimated at 2.7–10.7% with most women being asymptomatic with uneventful pregnancies [7–10]. However, leiomyomas can manifest during pregnancy with pain and discomfort due to torsion or degeneration. They have also been associated with early pregnancy failure as well as with obstetric complications such as preterm labor, abnormal fetal lie and presentation, placental abruption, post-partum hemorrhage and retained placenta [11].

The purpose of our study was to describe rate of leiomyoma growth in the 3 trimesters of pregnancy.

## Methods

We conducted a retrospective cohort study. Data were collected between January 2012 and December 2016 from the database of one of the main Israeli health maintenance organizations (Maccabi Health Services). The study was approved by the institutional ethical review board and no informed consent was required.

Included in the study were women over the age of 18 who underwent routine US exam during pregnancy and who were diagnosed with at least one uterine leiomyoma. All women had at least two measurements during two different trimesters. Measurements included at least a bi-dimensional estimation of the leiomyoma size (mm). Excluded were women who failed to meet all inclusion criteria.

First trimester was defined up to 11 + 6 weeks gestation, 2nd trimester between 12 and 23 + 6 weeks gestation and the 3rd trimester between 24 + 0 weeks and date of delivery.

Demographic data collected included maternal age, parity, body mass index (BMI) and gestational age during the US exams. Leiomyoma characteristics included length and width in (mm) and area (length X width). Number of leiomyomas, location (right, left, anterior, posterior, fundal and cervical) and topographic site (sub-serosal, intramural and sub-mucosal) were also evaluated. In women who had multiple leiomyomas follow up was performed individually for each leiomyoma according to its original location.

The primary outcome was uterine leiomyoma growth pattern along pregnancy. Secondary outcomes included correlation between different parameters investigated and the percent of weekly or overall change in area of leiomyomas between trimesters.

Evaluation of uterine leiomyoma size included the following comparisons: (1) Size in the 1st trimester compared to 3rd trimester; (2) 1st trimester compared to 2nd trimester; (3) 2nd trimester compared to 3rd trimester and (4) 3rd trimester compared to 6 weeks post-delivery (available only for a subset of patients).

US exams were performed by either US technicians or physicians with expertise in performing such diagnostic studies.

Leiomyoma size was approximated by calculating the area of each tumor (length in mm X width in mm). Change in leiomyoma area was calculated as the difference between the latest area measurement and the initial area measurement, both in mm^2^. The growth rate per week, presented as increase in percent, was calculated using the following formula: [100 X (Change in area) / (Initial area)] / (Interval in weeks between measurements) [7].

### Statistical analysis

To assess the change between the two time points paired t-test, the one sample t-test and the Wilcoxon signed ranks test were applied. The Pearson Correlation Coefficient and the Spearman Non Parametric Correlation Coefficient were used to assess the strength of the association between two quantitative variables.

Comparisons of quantitative variables between 2 and 3 independent groups were carried-out using the Mann-Whitney non parametric test and the non-parametric Kruskal-Wallis test, respectively. All tests applied were two tailed and a *P*-value of 0.05 or less was considered statistically significant.

## Results

During the study period, 377 patients with 458 uterine leiomyomas were evaluated sonographically. After applying our inclusion criteria, 196 patients with an overall number of 248 leiomyomas were included in the analysis.

**Fig. 1:**
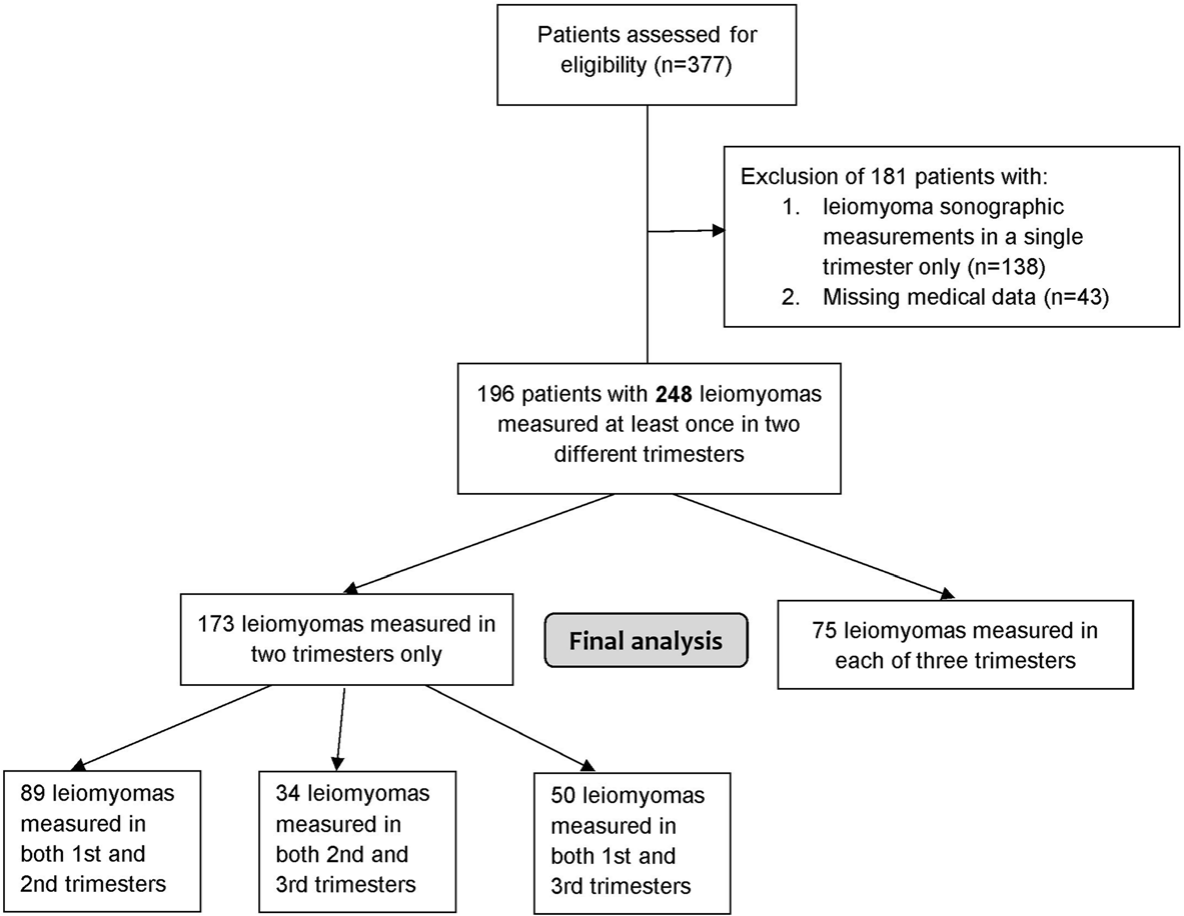
Flow chart of patient inclusion for final analysis

Of the 248 leiomyomas, 173 were assessed at least once in two different trimesters of gestation and 75 leiomyomas were evaluated at least once in each of three trimesters of gestation. A flow chart depicting the study population is presented in Fig. 1.

Basic characteristics of the study population and leiomyoma location are presented in Table 1. Table 2 shows the change in area of leiomyomas between the 1st and 2nd trimesters and 2nd and 3rd trimesters. The majority of leiomyomas increased in size between the 1st and 2nd trimesters (*p* < 0.01). Change in growth of leiomyomas between the 2nd and 3rd trimester did not reach statistical significance. Initial leiomyoma size was not found to have an effect on growth during pregnancy.

**Table 1:** Basic characteristics of the study population (196 women with an overall number of 248 leiomyoma)

Parameter	n(%) or mean ± SD
Age (years) (Range)	35.90 ± 5.07 (20–53)
Body Mass Index (BMI) (Range)	28.33 ± 5.91 (16–47)
Gravidity	2.52 ± 1.93
Parity	0.98 ± 1.45
Multiple gestation pregnancy	16 (6.5%)
Leiomyoma location	
Posterior	75 (30.2%)
Anterior	79 (31.9%)
Right	34 (13.7%)
Left	22 (8.9%)
Fundal	23 (9.3%)
Cervical	15 (6.1%)
Leiomyoma topographic location^a^	
Subserosal	88 (51.8%)
Intramural	68 (40.0%)
Submucosal	14 (8.2%)
Leiomyoma length pre-conception (mm)	44.91 ± 20.07
Leiomyoma width pre-conception (mm)	38.48 ± 17.05

^a^data regarding topographic location was available for 170/248 leiomyomas

**Table 2:** Modifications in leiomyoma area throughout gestation in patients with at least one measurement in two different trimesters

	1st vs. 2nd trimester	2nd vs. 3rd trimester	1st vs. 3rd trimester
N of leiomyomas	164	109	125
Increase in size	130 (79.3%)	55 (50.4%)	100 (80.0%)
Decrease in size	33 (20.1%)	53 (48.6%)	24 (19.2%)
No change	1 (0.6%)	1 (9.2%)	1 (0.8%)
Mean change (%)	54.54 ± 75.87%	17.88 ± 59.71%	95.90 ± 191.31%
*P* value^a^	0.007	NS	< 0.001

Data presented as n(%) or mean ± SD

^a^Statistical significance of the mean change (%) in size between trimesters

Seventy-five leiomyomas were measured at least once during each of the three trimesters of gestation. In this subgroup, the overall increase in leiomyoma area between 1st and 2nd trimesters was 60.62% ± 58.37% (*p* < .001) and the overall increase in leiomyoma area between 2nd and 3rd trimesters was 8.2943.57 ± % (*p* < .001). The percent of change in area throughout the 1st and 3rd trimesters was 74.13 ± 109.59% (*p* < .001).

A further analysis, assessing change in area per week, has shown a weekly increase in size of leiomyomas of 6.90% ± 6.12% (*p* < .001) between 1st and 2nd trimesters and a 3.10% ± 0.39% between 2nd and 3rd trimesters (*p* < .05).

All 54 leiomyomas measured in the 3rd trimester and 6 weeks post delivery decreased significantly. The average reduction in size was 44.25% ± 36.05% (*p* < .05).

We further analysed the change in size between gestational trimesters per specific location (posterior, anterior, right, left, fundal, cervical) and topographic site (sub-serosal, intramural, and sub-mucosal). A significant increase in leiomyoma area was demonstrated between the 1st and 2nd trimesters for location and topographic site. A significant increase in leiomyoma area was also demonstrated between the 2nd and 3rd trimesters for the anterior and right leiomyomas and in sub-serosal and intramural topographic sites. Statistical significance was lost when growth was analyzed per week.

Cervical leiomyomas assessed in the different trimesters showed a significant weekly and overall increase in size between the 1st and 2nd trimesters (7.63 ± 6.28% and 94.22 ± 70.25%, respectively; *p* < .05). However, this trend subsided between the 2nd and 3rd trimesters.

No correlation was found between age, gravidity, parity, BMI, number of leiomyomas, location and topographic site of leiomyomas and the percent of weekly or overall change in area of leiomyomas between trimesters (Table 3).

**Table 3:** Correlation (r) between basic parameters and the percent of change in size of the leiomyomas

	Overall change in area		Weekly change in area	
	1st to 2nd trimester	2nd to 3rd trimester	1st to 2nd trimester	2nd to 3rd trimester
Age	−0.063	−0.143	−0.086	−0.155
Gravidity	−0.066	− 0.034	− 0.091	− 0.065
Parity	− 0.101	− 0.008	− 0.14	−0.041
BMI	0.025	−0.022	0.01	−0.072
No. of leiomyomas	−0.07	0.149	−0.06	^a^0.219
Pre-gestational size	−0.15	−0.254	− 0.227	−0.224
Size at end of puerperium	0.039	−0.234	−0.048	− 0.275

^a^*P* value =0.024. However, this correlation did not remain significant after adjusting for multiple comparisons

## Discussion

Uterine leiomyomas are a relatively common finding during pregnancy. While often asymptomatic, leiomyomas have the potential of becoming clinically important during pregnancy, depending on size and location [7]. Over the years an attempt has been made to describe the natural history of uterine leiomyomas during pregnancy. Previous studies have presented equivocal data with some studies describing increase in leiomyoma size during pregnancy while others report no change or decrease in size [7, 12–15].

In our study we found that uterine leiomyomas have a distinct growth pattern during pregnancy. We have shown that while leiomyomas increase in size during pregnancy, their growth pattern is differential with a substantial increase in size between the 1st and 2nd trimesters that is later attenuated.

Our results correlate partially with data published in previous studies. In their systematic review of the literature Vitagliano et al. reported on distinct leiomyoma growth between the 1st and 2nd trimesters with contradictory evidence with respect to 3rd trimester growth [12]. De Vivo et al. showed an increase in leiomyoma size between the 1st and 2nd trimesters as well as between the 2nd and 3rd trimesters [7]. In another series, Aharoni et al. presented leiomyomas to be mostly unchanged during pregnancy (59%). In cases where increase in size was noted (22%) growth percentage was minor (25%) [13]. In a series of 137 leiomyomas in 72 women Neiger et al. found similar results with no change in leiomyoma size during pregnancy [14].

In our study the initial leiomyoma size was not found to have an effect on growth during pregnancy. Contrary to our findings Lev-Toaff et al. followed 113 pregnant women with uterine leiomyoma while taking into account their original size. They showed that small leiomyomas grew during the 1st and 2nd trimesters but reduced in size during the 3rd trimester as opposed to large leiomyomas which grew during the first trimester but shrunk during the 2nd and 3rd trimesters [15].

Previous studies showed a possible association between leiomyoma growth and multi-parity, pre-pregnancy body mass index and maternal age [7]. Multivariate analysis performed in this study did not find any linkage between leiomyoma growth and any of the parameters investigated.

Full understanding of the pathogenesis of uterine leiomyoma is still lacking but clearly sex steroid hormones play a major role in their growth process [2, 3]. However, estrogen and progesterone are unlikely to be the sole actors involved. Estrogen and progesterone serum levels peak during the 3rd trimester of pregnancy while in most studies the major increase in leiomyoma size occurs in the 1st trimester [7]. This has led researchers to search for other proteins and hormones that might affect leiomyoma growth during pregnancy.

A large body of evidence has accumulated in favor of HCG as a possible stimulator of leiomyoma growth [5, 6]. This stimulatory effect seems to be mediated by the leuteinizing hormone/HCG receptor complex. This complex was shown to be expressed in myometrium as well as in leiomyomas in animals and humans [16]. It is postulated that these receptors are sensitive to exponential rise in HCG levels. As pregnancy advances down regulation of these receptors stunts leiomyoma growth leading to a decrease in leiomyoma size [16].

In our study cervical leiomyomas, which can affect mode of delivery, had the highest rate of increase in size compared to leiomyomas in other locations. Data regarding cervical leiomyomas is limited. Tian et al. described a series of 17 women with cervical leiomyoma during pregnancy. Sixteen of those women were delivered by cesarean delivery, mostly due to obstructed labor, and three underwent hysterectomy at the time of the cesarean section or shortly after due to post-operative complications. A statistically significant correlation was found between blood loss volume and leiomyoma size [17]. These data suggest an association between large cervical leiomyomas and adverse outcomes during delivery but clearly this is a topic in need of further investigation.

Besides its retrospective nature, this study has certain limitations. Information regarding ethnicity was unavailable. Size of uterine leiomyomas was estimated using area as opposed to volume. This was due to lack of data regarding a third dimension in most of the US exams performed. US exams were performed by different ultrasound technicians as well as by physicians, possibly leading to operator bias. However, as all exams were performed by the same health provider, the same protocol was used for all exams. Uterine leiomyomas were measured during different gestational weeks. This was dealt by adjusting the results per week of gestation and calculating growth per week of gestation. The number of women included in our study constitutes one of the largest series presented to date on this topic. In the future, well planned prosepective studies focusing on women diagnosed with uterine leiomyomas during pregnancy should be able to address most of these limitations.

## Conclusions

The main finding of our study is that uterine leiomyomas grow substantially during the 1st trimester of pregnancy but this trend is attenuated later in pregnancy with minimal growth towards the end of gestation. Further and prospective studies are needed to characterize change in leiomyomas during pregnancy and their clinical importance.

## Data Availability

The datasets used and/or analyzed during the current study are available from the corresponding author on reasonable request.

## Abbreviations

BMI: Body mass index
HCG: Human chorionic gonadotropin
US: Ultrasound
